# Narcissistic Personality Disorder: Trends in Communities and Prison Populations, and Its Association with Criminal Behavior

**DOI:** 10.3390/bs16060986

**Published:** 2026-06-13

**Authors:** Barbara Gawda

**Affiliations:** Department of Psychology of Emotion & Personality, Maria Curie-Sklodowska University, 20-612 Lublin, Poland; bgawda@wp.pl

**Keywords:** worldwide populations, narcissistic personality disorder, prevalence, prisoners, violent crimes

## Abstract

This article aims to discuss worldwide trends in the prevalence of narcissistic personality disorder (NPD) among prisoners compared to community samples. We also aim to show how this disorder is associated with criminal behavior and types of offenses. The results of the literature review document a relatively low and stable prevalence of narcissistic personality disorder compared to the frequency of other specified personality disorders in many countries worldwide. The results suggest that the rates of narcissistic personality disorder among prisoners in many countries are higher than those in communities. It has been found that this disorder is associated with domestic violence and other violent criminal behaviors, particularly with fraud and forgery violations. It has also been shown that offenders with narcissistic personality disorder are perceived as less guilty. Furthermore, research on the treatment of offenders with narcissistic personality disorder is sparse, which indicates that the treatment of NPD is limited, and it poses a challenge for mental health professionals as well as those who work in the penitentiary system.

## 1. Introduction

Although NPD has long been included in diagnostic classifications, and there are many common beliefs about its increasing prevalence worldwide, there is a scarcity of research based on representative samples that could confirm this increase. There is a need to deepen our understanding of NPD, particularly because it is associated with a wide range of mental health issues and criminal acts. This review aims to describe the prevalence of NPD and its relationship with criminal behavior. Research on the relationship between personality disorders and criminal offenders has mainly focused on antisocial personality disorder, which is the most prevalent personality disorder in offender populations (Fazel & Danesh, 2002). However, other personality disorders from Cluster B, such as narcissistic personality disorder (NPD), are significantly associated with criminal behaviors (Young et al., 2018). Narcissistic personality disorder is underrated in this context; it is the third most common in the prison population. NPD has been examined in a few studies that link this disorder with crimes against others, homicide, fraud, and forgery (Jones & Paulhus, 2010). Offenders with NPD were described as motivated by the need to exert power, domination, and control over their victims, which is consistent with the calculated and controlling behavior in the course of some fraud offenses. Criminals with NPD are perceived by the court as less guilty because they make an impression of suffering and unhappiness. An example of a criminal with narcissistic personality disorder is Patrizia Reggiani (she was diagnosed with NPD), who ordered the murder of her ex-husband Maurizio Gucci. She was sentenced to 29 years in prison for arranging the killing, and she was released much earlier after 18 years in prison. This case illustrates the links between crime and NPD. Scientists have pointed out that there are particular associations between higher IQ and narcissistic personality among offenders. NPD is associated with life success in terms of career and finances (Jones & Paulhus, 2010).

It is worth comparing worldwide data on the prevalence of NPD. When analyzing the clinical form of narcissism, it is important to highlight that the diagnostic status of the DSM narcissistic personality disorder as a specified personality disorder has been controversial for decades. NPD is far from being a well-established gold standard for narcissism. This personality disorder was present in earlier classifications of mental disorders, that is, in the DSM-3 (Diagnostic and Statistical Manual of Mental Disorders-3rd ed.) and the DSM-4. It has also been included in the latest version, the DSM-5. The definition of this disorder in the DSM-5 is similar to the former classifications, even identical to the DSM-4 (American Psychiatric Association, 2000). According to the DSM-5 (American Psychiatric Association, 2013), narcissistic personality disorder (NPD) is defined as “an enduring pattern of behavior and emotional response related to the sense of superiority, need for admiration, lack of empathy, beginning in early adulthood and present in a variety of contexts, as indicated by at least five of the following: has a grandiose sense of self-importance (e.g., exaggerates achievements and talents), is preoccupied with fantasies of unlimited success, power, brilliance, beauty, etc., believes that he or she is ‘special’ and unique, requires excessive admiration, has a sense of entitlement, is interpersonally exploitative, that is, takes advantage of others to achieve his or her own ends, lacks empathy; is unwilling to recognize or identify with the feelings and needs of others, is often envious of others or believes that others are envious of him or her, shows arrogant, haughty behavior or attitudes”(p. 423) (Butcher et al., 2016). The diagnosis of NPD is complex, not only because of imprecise criteria but also because of its manifestation in different forms. Its subtypes are grandiose and vulnerable narcissism. The grandiose subtype reflects traits related to grandiosity, aggression, and dominance. Individuals who demonstrate this type of narcissism have a sense of entitlement and uniqueness, continuously speak about their achievements, and crave admiration. However, the vulnerable type has not been described in detail. Clinicians point out that such persons are particularly fragile, emotionally unstable, excessively shy, preoccupied with fantasies of success, and at the same time ashamed of such ambitions. According to the five-factor model, there are key differences despite a number of similarities; grandiose narcissists demonstrate a low level of some components of neuroticism and high extraversion, while vulnerable narcissists are described as embittered, intolerant, cruel, extremely tense, and worrying. Furthermore, diagnosis can be more complicated because narcissistic individuals may fluctuate between grandiose and vulnerable presentations (DSM-5, American Psychiatric Association, 2013, p. 424).

## 2. Methods

To discuss the trends of NPD in modern societies and prisons, worldwide data on the occurrence of this disorder are described. Data on the prevalence of NPD come from epidemiological research (Tyrer et al., 2015). A web database search, including PsycInfo, PsycARTICLES, PubMed, PubPsych, Scopus, Web of Science, and Google Scholar, was used to present prevalence data. We used a combination of keywords: prevalence, narcissistic personality disorder, community, prisoners, and criminal behavior. The aim was to find publications with data on the prevalence of NPD. This search resulted in many findings, which were narrowed down to 39 articles/chapters that were qualified to be included in the analysis. The publications cover the period from the 1980s to 2025. All research has been analyzed; however, the final conclusions are based on 16 selected studies that were conducted using the same tool, identified personality disorders with standardized diagnostics according to the DSM-4/DSM-5 or ICD-10, and had a sufficiently large representative sample (Table 1). Some studies were also not included in the final analysis as they did not report the prevalence in percentage but rather the means of narcissistic personality disorder traits (for instance, Rossier et al., 2013). Some articles published in the 90s in the USA were excluded to avoid repeating information about the US population. The complete list of examined research is presented in Table A1. This table also contains information on the country, sample size, type of sample, tools used in the diagnosis of NPD, percentage of NPD/or means of NPD traits, and whether the study was considered in the final conclusions. All studies listed in Table A1 with the annotation “no” were excluded from the final conclusions due to sample sizes (too small or unrepresentative) or the use of older or other diagnostic tools than the DSM-4 or DSM-5.

This review is based on numerous references not included in Table 1, as many do not offer data regarding the prevalence of NPD in the general population or among incarcerated individuals. Nonetheless, they include different information about the occurrence of other personality disorders, criminal behaviors associated with NPD or personality disorders that co-exist with NPD. Table 1 includes only articles that fulfilled the criteria: specifically, the article provided data on the prevalence of NPD in the general population or among prisoners, involved a large representative sample, and utilized DSM-4/DSM-5/ICD-10 tools.

## 3. The Prevalence of NPD Worldwide in the General Population and in Prisons

The goal of this article is to discuss and compare the worldwide trends in the prevalence of NPD in community and prisoner samples, as well as to present its relationship with criminal behaviors and the possibilities of treatment of NPD.

**The USA**. Most research on the prevalence of NPD has been conducted in the United States (Newton-Howes et al., 2015). This data shows that, for instance, based on research published in 1989 and 1993, the most frequently occurring disorder is obsessive–compulsive disorder, and no NPD was reported (Black et al., 1993; Zimmerman & Coryell, 1989). In other publications, the prevalence of NPD was estimated to be 0.4% (Reich & de Girolamo, 1997). In turn, a survey conducted on 229 respondents with the PDE technique from New York and published in 1995 revealed that NPD was observed in 3.9% of the relatives of normal controls (Klein et al., 1995). In a survey conducted on a larger population of students from Ithaca published in 1997 using the IPDE technique, it was revealed that the NPD prevalence was 2.7% (Lenzenweger et al., 1997). In turn, research conducted on a community sample from Baltimore demonstrated that only 0.03% of the sample may show traits of NPD (Samuels et al., 2002). In 2004, wide-ranging epidemiological studies conducted on a sample of more than 4300 Americans using the DSM-4-based tool showed that obsessive–compulsive disorder had the highest prevalence, whereas NPD was not documented at all (Grant et al., 2004). Another study examined the New York community and presented a rate of 2.2% for narcissistic personality disorder (Crawford et al., 2005). Later, Lenzenweger et al. (2007), based on the screening with the IPDE technique, found no instances of NPD among community participants. In the following years, the NESARC was replicated, which examined a large community sample (n = 34,653). This survey revealed that obsessive–compulsive personality disorder is highly prevalent, while NPD is prevalent in 6.2% of the population (Grant et al., 2004; Pulay et al., 2009; Stinson et al., 2008). Overall, the occurrence of NPD in the US population is lower than the mean prevalence of any personality disorder (14.8% prevalence). Using a different algorithm to calculate the prevalence of disorders in the revised version of NESARC, it was determined that the prevalence of NPD was 1% (Trull et al., 2010). NESARC wave 3 was conducted on a large community sample in 2012–2013 (N = 36,309). No specific data on NPD in the U.S. were provided. The mean prevalence of NPD in Western countries, including the USA, is 1.23% (Volkert et al., 2018). In contrast, research on the prevalence of NPD among prisoners showed that this disorder is diagnosed in 7.8% of inmates (Young et al., 2018). It is worth noting that although the general rates of Cluster B personality disorders increased between 1980 and 2016 in USA prisons, the rate of NPD was stable and did not change over the years (Jones & Paulhus, 2010).

**Other North American countries.** The analysis of other North American countries, such as Mexico, shows a generally lower prevalence of personality disorders than that in the USA. In a national survey on the occurrence of disorders in Mexico, which was conducted on a large sample (n = 2362), it was demonstrated that the highest prevalence was for Cluster A disorders (4.6%), followed by Cluster C (2.4%), and the lowest prevalence was for Cluster B disorders (1.6%) (Benjet et al., 2008). NPD is classified in Cluster B; however, exact data on NPD are not available. These data suggest that NPD is rarely reported in the Mexican population. With regard to Canada, it is difficult to address the issue due to the lack of data on NPD apart from community data on personality disorders. In general, the prevalence of personality disorders in Canada is similar to that in the USA (Stewart et al., 2002).

**South American countries.** Data regarding South America are also scarce. The general prevalence of personality disorders in Colombia is similar to that in Mexico (B. Huang et al., 2006). In Brazil, research has shown that among students, NPD is the third most frequent (average of 2.19%). This group was not representative of the Brazilian population. The most frequent are borderline and obsessive–compulsive disorders (Bartholomeu et al., 2015). Another study conducted on a large community sample in Brazil revealed that 63.5% of participants screened positive for personality disorders; however, the authors did not report data on NPD (Pianowski et al., 2024). It is worth noting that people from South America may be misdiagnosed, particularly with regard to borderline and histrionic disorders. This is because the typical traits of both disorders, such as hyper-emotionality, somatization, tendency to dramatize, and seductiveness, are typical means used for expressing emotions in populations in these regions. Similarly, in Southern European cultures, where other social norms are accepted for expressing emotions, intense and excessive expression can be misdiagnosed as symptoms of personality disorders. This also applies to the diagnosis of NPD in these cultures/countries, which is somehow linked to such a type of emotional expression (Calliess et al., 2008).

**European countries**. The general prevalence of personality disorders in Europe is closer to that in the USA than in Latin America (Y. Huang et al., 2009). Based on research conducted using the DSM-4 tool, it was determined that in Great Britain, the most frequent Cluster C disorder is obsessive–compulsive disorder; however, no cases of NPD were reported in the analyzed sample of 626 people (J. Coid et al., 2006). Among prisoners in the UK, the mean NPD trait score is 3.19 (Roberts & Coid, 2010). Blackburn and Coid (1999) report the high rate of NPD among UK violent offenders (55%). A study conducted in the Netherlands among mentally ill prisoners (n = 5257) using the DSM-5 and DSM-4 measures revealed that the prevalence of NPD is 0.9% (van Buitenen et al., 2020). It should be noted that this group (mentally ill prisoners) also includes a small sample of Cluster B personality disorders. The study by de Ruiter and Trestman (2006) presents statistics on mental health disorders among prisoners in Denmark but does not include data on NPD.

The data on the prevalence of personality disorders in Norway indicated that in the Oslo population, disorders occur in 13.4% of the population, and NPD occurs in 0.8% (Torgersen et al., 2001). Among Norwegian prisoners, the NPD rate is higher at 9.4% (Cramer, 2016). In Sweden, the overall prevalence of personality disorders is also high; obsessive–compulsive disorder is considered to be the most frequently occurring disorder, and NPD is reported at a rate of 2.9% (Ekselius et al., 2001). In Romania, a study in a maximum-security prison was conducted, and among 60 prisoners, 35% met the criteria for NPD (Sabareanu & Gonta, 2023). In Austria, the community prevalence of narcissistic personality disorder was estimated to be 2.0% (Loranger et al., 1997). In Germany, obsessive–compulsive disorder is considered the most frequently occurring personality disorder (4,4%), whereas NPD is lower at 3.5% (Loranger et al., 1997). In Switzerland and Luxembourg, no cases of NPD in 1997 were diagnosed (Reich & de Girolamo, 1997). It is important to note that the research presented by Loranger and associates in 1997 and by Reich and de Girolamo in the same year was based on a small sample size and was inconclusive. Therefore, this research is not included in Table 1, which summarizes the appropriate data on the prevalence of NPD in communities and prisons. Similar data have been reported for the Spanish community. The Spanish prisoners present a varied occurrence of NPD: 2.0% according to Arroyo and Ortega (2009) and 21.08% according to Flórez et al. (2019). In Portugal, a study on a sample of 294 prisoners was conducted, and the prevalence of NPD was established to be 35% (Brazão et al., 2015).

Data on the community occurrence of personality disorders in Poland were obtained from two sources. The first one is a non-clinical sample survey with the use of an electronic version of the questionnaire Lifestyles 05/F conducted by Trzebińska and Gabińska (2014). Surveys have demonstrated that the prevalence of NPD is 4.7% (Trzebińska & Gabińska, 2014). The other source is research using the Structured Clinical Interview for DSM-4 Axis II Disorders (SCID-II), conducted on a large sample (n = 1460) selected from the general population (Gawda & Czubak, 2017). Narcissistic personality disorder traits were frequently observed in the Polish population (7.00%). This is consistent with other findings on the Polish population, which also revealed an increased level of covert narcissism compared to the Dutch population (Zondag et al., 2009). The data on NPD among Polish prisoners showed that the prevalence of narcissistic personality disorder traits was 4.8 (Czubak & Gawda, 2020). This study determined the high level of personality disorder traits.

**Middle East and Asia.** Data from Asian countries, such as Turkey, were represented by single reports. A survey of the general Turkish population using the DSM-4 and ICD-10 (1992) techniques (DIP-Q) revealed that at least 20% of the population showed symptoms of personality disorders. The most frequent disorders mentioned by the authors were schizotypal and obsessive–compulsive, whereas NPD rates were, on average, two times lower and/or not identified (Dereboy et al., 2014). Among prisoners convicted of murder in Turkey, the NPD rate was established as 4.3% based on the examination of more than 500 incarcerated murderers (Kugu et al., 2008). Based on surveys of Asian populations, narcissistic personality disorder is rare. For example, the prevalence of NPD in the Japanese population is estimated to be 1.8% (Loranger et al., 1997). Overall, in Asian countries (e.g., China and India) or among people of Asian origin, the prevalence of personality disorders is lower than that in North American and Western European countries (Gupta & Mattoo, 2012; Y. Huang et al., 2009; Tang & Huang, 1995; Yang et al., 2000). The prevalence of “other personality disorder, including NPD and self-defeating” (term used in the manuscript by Gupta and Mattoo) among Indian outpatients is 5.2%; however, the exact data on community prevalence and among prisoners is not available (Gupta & Mattoo, 2012). Some disorders, such as avoidant or dependent disorders, do not appear in the Chinese Classification of Mental Disorders. NPD diagnosis has rarely been used in the clinical practice of psychiatry in China, and the frequency of NPD in the Chinese clinical or prison populations is largely unknown. However, recent research has shown that the frequency of NPD in an outpatient counseling setting is 4.0% (Jiang et al., 2019). The low rate of NPD is interpreted as being connected to the strong influence of Confucian philosophy on the mentality and behavior of the Chinese population (Loranger et al., 1997). Interpersonal relationships are based on values derived from this philosophy, such as good manners, integrity, and forgiveness. On the other hand, the values appreciated in Japan, such as high perfectionism and strong commitment to precision, order, and accuracy, may influence the functioning of individuals in such a way that they create favorable conditions for developing obsessive–compulsive behaviors, which results in a great intensity of such traits in the Japanese population (Loranger et al., 1997).

**African countries**. Data for the overall prevalence of personality disorders in Africa are represented by single reports (Y. Huang et al., 2009). Narcissistic personality disorder was not diagnosed in the study by Loranger et al. (1997). Some studies do not report data regarding this disorder (Y. Huang et al., 2009). The first large-scale study of personality disorders in African cultures was conducted, including participants from countries such as Algeria, Tunisia, Benin, Burkina Faso, Mali, Senegal, the Democratic Republic of the Congo, the Republic of the Congo, and Mauritius (Thuo et al., 2008). It confirmed the cross-cultural applicability of the International Personality Disorders Examination (IPDE) and showed that the DSM-4 Axis II PDs’ taxonomy may be useful in different cultural settings (Rossier & Rigozzi, 2008). Then, studies on PDs in urban and rural regions of Burkina Faso conducted in a large sample (1750 participants) showed mild NPD traits (see Table A1). However, the authors emphasize that the means cannot be interpreted because the PD scales did not reach scalar equivalence. The authors used the self-measure screening French and Mooré versions of the IPDE (Rossier et al., 2013). Furthermore, the authors highlighted that translating a complex psychological questionnaire into native African languages is a highly sophisticated task (Rossier et al., 2013). In prisons in African countries, many mental disorders are diagnosed; the rates of NPD show that this disorder is rare in Egypt at 0.10% (El-Gilany et al., 2016). In Kenya, a study involving a sample of 325 inmates (both sexes) was conducted. It was found that 68.8% of prisoners met the criteria for NPD. The authors concluded that more than two-thirds of the Kenyan prison population exhibits narcissistic personality traits. They also established that NPD is significantly more prevalent than ASPD (antisocial personality disorder) in Kenyan prisoners, and that 17.9% of prisoners present dual diagnoses, i.e., both NPD and antisocial personality disorder (Ngunjiri & Waiyaki, 2025).

**Australia**. The Australian population was surveyed for the occurrence of personality disorders, and the general rate was estimated. However, NPD, in contrast to obsessive–compulsive disorder, was not identified (Jackson & Burgess, 2000). Another study among Australian women revealed a tendency for an overall increased occurrence of personality disorders, a lower than 1.7 percent frequency of NPD, and, in contrast, a higher occurrence of obsessive–compulsive personality disorder (Quirk et al., 2017).

## 4. Discussion

### 4.1. Conclusions on the Prevalence

In order to draw accurate conclusions, we limited the final conclusions to data based on comparable criteria, i.e., the DSM-4/5 techniques, large sample sizes, general representative population samples in the case of NPD general prevalence, and large and appropriate (representative) prisoner samples in the case of NPD prevalence in prisons. When we apply these criteria, only 16 studies can be included in the final analysis. They are listed in Table 1. In general, the data on the prevalence of NPD documents that NPD is relatively cross-culturally similar and temporally stable from the 1980s through 2020 and does not show an increase (McGilloway et al., 2010). We cannot analyze the temporal trend in the prevalence of NPD in countries other than the USA due to the scarcity of appropriate data. These results are in line with cross-cultural studies indicating that the structure and prevalence of NPD are stable across cultures, as presented by Dhawan and associates (Dhawan et al., 2020).

Referring to the thesis that individualistic cultures are linked to increased narcissism, it is important to note that the selected studies were conducted in different cultures in terms of individualism vs. collectivism (Green et al., 2005). Some of these studies were conducted in Western cultures that are considered individualistic, i.e., the USA, UK, Norway, Sweden, Poland, and Spain, while three studies were conducted in African and Asian cultures that are considered collectivistic (Egypt, China, and Turkey [Turkey has been described in the literature as a collectivist country by Dereboy et al., 2014). The community prevalence rates cover the years from 1997 to 2020, and they range from 0.0–6.2% in the USA to 7.1% in Turkey. In the USA, the prevalence rates for NPD are slightly lower than in Turkey (a collectivist country) or in Poland. We can therefore conclude that the reported results on NPD occurrence do not confirm the thesis that individualist/Western/modern cultures are associated with increased narcissism. The prevalence of this disorder is reported to be much lower in populations than, for instance, obsessive–compulsive personality disorder or other specified personality disorders (Sansone & Sansone, 2011; Tyrer et al., 2010). The mean prevalence of NPD in Western countries is 1.23% (Volkert et al., 2018).

Our comparisons have revealed that the occurrence of NPD is significantly higher among prisoners compared to the general population in various countries. For instance, while the prevalence of NPD in Egyptian prisons is 0.1%, it is as high as 9% in Norwegian prisons. This disparity could potentially be attributed to cultural differences. It is important to note that even a small percentage of individuals with NPD can have a significant impact on society as a whole (J. W. Coid, 2002). This personality disorder is associated with a heightened risk of serious criminal behavior (Maples et al., 2010; Martinez et al., 2008). Furthermore, the prevalence of NPD in prison populations is often accompanied by other psychiatric disorders, which are more prevalent among prisoners compared to the general population (Fazel & Danesh, 2002). In fact, personality disorders are a common mental health issue observed in prisons.

### 4.2. NPD and Criminal Behavior

In criminal populations, NPD is more commonly diagnosed in men, with 50–75% of patients being male compared to females. Research has shown that NPD is the third most prevalent personality disorder among prisoners and is often associated with certain types of crimes, such as homicide, fraud, and forgery (Young et al., 2018). Prisoners with NPD exhibit specific patterns, including fragile egos and a tendency to feel deeply hurt by criticism (DSM-5, American Psychiatric Association, 2013; ICD-10, 1992). Thus, treatment for NPD often involves managing feelings of anger, depression, and shame through psychotherapy (Kraus & Reynolds, 2001). According to some researchers, NPD is associated with “white-collar” crimes. In fact, 35% of “white-collar” criminals have been found to meet the criteria for NPD (Blickle et al., 2006). Recent data also suggest that “red-collar” criminals, who exhibit traits of psychopathy and entitlement, are often diagnosed with NPD as well. These individuals may use violence to silence those who uncover their fraudulent activities (MacDonald, 2022). Fraud offenses require specific skills, according to Roberts and Coid (2010). The high IQ of individuals with NPD may contribute to their success in committing financial fraud (Young et al., 2018). The criminals with NPD are portrayed as being motivated by the need to exert power, domination, and control over their victims. They present manipulative, calculative, and controlling behaviors in the course of some fraud offenses. Furthermore, they are perceived by the court and by society as less violent, which can have an impact on the interpretation of the criminal act and result in a milder sentence (Young et al., 2018). They may also display characteristics such as perfectionism, shame, and maladaptive aggressiveness. These traits are interconnected in individuals with NPD, as perfectionism can lead to feelings of shame and aggression when their high standards are not met (Fjermestad-Noll et al., 2020a). This perfectionism may be self-oriented or other-oriented, with the latter being specifically linked to narcissistic disturbances (Stoeber, 2014). Prisoners with NPD may also impose their perfectionistic standards on others and become aggressive when these expectations are not met. This can lead to violent crimes, as narcissistic individuals may feel threatened by any signs of failure (Nealis et al., 2016).

Another feeling often presented by individuals with NPD is shame. It can be linked to feelings of helplessness, impotence, weakness, and loss of control. Omnipotence and rage can therefore act as effective psychological defenses against feelings of shame. If shame is mobilized by narcissistic injury, it can activate aggressive behavior rather than induce depression, as one may be compelled to counterattack (Velotti et al., 2014). Thus, maladaptive aggression is often expressed by prisoners with NPD. Krizan and Johar (2015) found that anger and hostility arise from threats to the narcissistic self-image, and that, in particular, persons exhibiting vulnerable narcissistic features are prone to both internalize and externalize anger, and that this is fueled by suspiciousness, dejection, and angry rumination. Individuals with NPD believe they are superior to others and that others should recognize that perceived superiority. Despite their feelings of superiority, individuals with NPD have “very fragile” self-esteem. The deficiencies in self-esteem make individuals with NPD especially vulnerable to criticism, even a little, and when they suffer from criticism, they may feel “humiliated, degraded, hollow, and empty,” which leads them to violence and crime (Fjermestad-Noll et al., 2020b).

Emotional impairments are the core traits of every personality disorder, showing up in various forms but all are rooted in neural organization. All personality disorders, including NPD, are deeply rooted in neural mechanisms. Because of this, patients with personality disorders are uncompromising in their behavior, emotions, and cognition. This presents a challenge for their treatment, as they are resistant to change, though long-term treatment can have positive effects (Moran et al., 2016).

All the above-described characteristics of individuals with NPD are crucial in the process of treatment and social rehabilitation. Researchers (Ciosek & Pastwa-Wojciechowska, 2016) believe that personality disorders in prisoners significantly reduce the effectiveness of social rehabilitation. First, it inhibits social rehabilitation; second, it has negative consequences for the functioning of inmates in prisons, causing self-destruction, faulty forms of adaptation, and other mental health problems (Szwejkowska, 2023; Wysocka, 2008). Not only are prisoners suffering from psychopathic personality disorder highly problematic for any routine practice in European and world prisons, but criminals with other personality disorders, such as NPD, are also troublesome (Fazel & Danesh, 2002). In addition, research on the treatment of this disorder in prisons is scarce (Dhawan et al., 2020; Weinberg & Ronningstam, 2020). The burden of treatment of serious mental disorders in prisoners is substantial. Thus, the lack of research in this area documents that the treatment of criminals with NPD is difficult and limited. It is a challenge for mental health professionals and those who work in the criminal justice system. Furthermore, the existence of many incarcerated persons in prisons with mental illnesses, even if they are not severe, contributes to behavioral maladjustment (Apostolopoulos et al., 2018; Weinberg & Ronningstam, 2022). This has a dysfunctional effect on the social climate within the prisons and limits the capacity to adjust to the setting. Such a social climate transmits stress to the prisoners and the prison staff members who are in contact with prisoners.

#### Limitations

Our study has some limitations. The comparisons here must be viewed with caution due to cross-cultural differences between the samples. To ensure accuracy, we limited our comparisons to data with comparable criteria, such as the use of DSM-4/DSM-5/ICD-10 techniques and large sample sizes.

## 5. Conclusions

There is still a lack of studies based on representative samples across multiple countries that would allow for an estimate of the global prevalence of NPD. Most studies come from the United States and Western countries. Regarding the prevalence of NPD, documentation indicates that this disorder shows considerable cross-cultural similarity and temporal stability from the 1980s to the 2020s. The empirical studies show that NPD scores have not increased over time. Some publications claim narcissism has increased, but cohort studies document its decline. The results contradict the claim that recent cohorts of college students are more narcissistic than earlier generations (Wetzel et al., 2017). The presented results are in line with cross-cultural studies indicating that the prevalence of NPD is low or average across different cultures. Research does not support the claim that individualistic cultures are associated with higher rates of NPD. The higher prevalence of NPD in prison populations co-occurs with other psychiatric disorders, which are substantially higher among prisoners than in the general population. Thus, NPD carries risks of having serious criminal problems and a variety of criminal acts, in particular, forgery, violation, fraud, and violence. Most of the available data from prisons are derived from Western countries. This fact emphasizes the need for greater forensic psychiatric research in non-Western populations (Fazel & Danesh, 2002).

## Figures and Tables

**Table 1:** Prevalence of NPD according to the studies on communities and prisoner populations.

Country	NPD %	Authors
	**Western Communities**	
USA	0.03	Samuels et al. (2002)
	2.00	Crawford et al. (2005)
	0.00	Lenzenweger et al. (2007)
	6.20	Stinson et al. (2008); Grant et al. (2008); Pulay et al. (2009)
Western countries (USA)	1.23	Volkert et al. (2018)
Norway	0.80	Torgersen et al. (2001)
Poland	7.00	Gawda and Czubak (2017)
UK	0.00	J. Coid et al. (2006)
Sweden	2.90	Ekselius et al. (2001)
	**African and Asian countries**	
China	4.00	Jiang et al. (2019)
Turkey	7.10	Dereboy et al. (2014)
	**Prisons**	
USA	7.30	Young et al. (2018) (from 1980 to 2016)
Norway	9.40	Cramer (2016)
Netherlands	0.90	van Buitenen et al. (2020)
Spain	2.00	Arroyo and Ortega (2009)
Egypt	0.10	El-Gilany et al. (2016)

## Data Availability

No new data were created or analyzed in this study.

## Abbreviations

The following abbreviations are used in this manuscript:

NPD	Narcissistic Personality Disorder
IPDE	The International Personality Disorders Examination
SCID-II	The Structured Clinical Interview for DSM-IV Axis II Disorders
DSM-3/DSM-4/DSM-5	Diagnostic and Statistical Manual of Mental Disorders-3/4/5th Edition
ICD-10	International Statistical Classification of Diseases and Related Health Problems
NESARC	The National Epidemiologic Survey on Alcohol and Related Conditions
SIDP	The Structured Interview for the DSM-3 Personality Disorders
PDQ-4	The Personality Diagnostic Questionnaire-4 by S.E. Hyler
PDI-IV	The Personality Disorder Interview-IV by T.A Widiger
IDTP	The Personality Disorders Dimensional Inventory by L. de F. Carvalho (version for homeless)

## Appendix A

**Table A1:** Studies on NPD in community and prisoners’ samples with information on sample type, sample size, and methods.

Study: Authors (Date)	Country	Sample Type	N	Method	NPD % or Means of PD Traits	Included or Not in the Final Conclusions
Communities						
Zimmerman and Coryell (1989)	USA	Iowa, relatives of patients, controls	797	SIDP DSM-3	0.0	no
Black et al. (1993)	USA	Iowa, relatives of OCD, controls	247	SIDP DSM-3	0.0	no
Klein et al. (1995)	USA	New York relatives controls	229	PDE DSM-3	3.9	no
Lenzenweger et al. (1997)	USA	Ithaca University students	1646	IPDE DSM-3-R	2.7	no
Samuels et al. (2002)	USA	Baltimore Community sample	742	IPDE, DSM-4	0.03	no
Grant et al. (2004)	USA	Community sample	43,093	Interview -DSM-4	-	yes
Crawford et al. (2005)	USA	New York Community sample	644	SCID-II DSM-4	2.0	yes
Lenzenweger et al. (2007)	USA	Community sample	5692	Screening IPDE	0	yes
Grant et al. (2008), Stinson et al. (2008), Pulay et al. (2009)	USA	Community sample	34,653	NESARC 2 DSM-4	6.2	yes
Trull et al. (2010)	USA	General population	40,000	NESARC Revised	1%	yes
Volkert et al. (2018)	Western countries (USA)	General population	36,309	NESARC 3 DSM-5	1.23%	yes
Bartholomeu et al. (2015)	Brazil	College students	250	IDTP	2.1 (M)	no
Loranger et al. (1997)	Austria	Vienna patients	50	ICD-l0 Screening IPDE	2.0	no
	Netherlands	Patients	65		0	
	Switzerland	Geneva patients	32		0	
	London	Patients	52		0	
	Luxembourg	Patients	52		0	
	Germany	Munich patients	113		3.5	
	Kenya	Nairobi patients	50		0	
	Japan	Tokyo patients	57		1.8	
Rossier et al. (2013)	Burkina Faso	Community sample	1750	IPDE Screening DSM-4	(a) 4.94F/5.05M * (b) 4.59F/4.89M * (c) 5.15F/5.58M *	no
Yang et al. (2000)	China	Patients, Non-patients	1926 166	PDQ-4 PDI-IV	3.76 * 3.08 *	no
Jiang et al. (2019)	China	Outpatients	1402	SCID-II DSM-4	4.0%	yes
J. Coid et al. (2006)	UK	Community sample	626	SCID-II DSM-4	0.0	yes
Torgersen et al. (2001)	Norway	Oslo community sample	2053	SIDP DSM-3-R	0.8	yes
Ekselius et al. (2001)	Sweden	Community sample	557	DIP- DSM-4/ICD-10	2.9	yes
Trzebińska and Gabińska (2014)	Poland	General population	3509	Lifestyle 05/FS Inventory	7.00	no
Gawda and Czubak (2017)	Poland	General population	1450	SCID-II DSM-4	7.00	yes
Dereboy et al. (2014)	Turkey	General population	- 774	DSM-4/DIP-Q ICD-10	7.10	yes
Jackson and Burgess (2000)	Australia	Community sample	10,641	IPDE ICD-10	-	no
Quirk et al. (2017)	Australia	Women community	768	SCID-II DSM-4	<1.7	no
Prisons						
Young et al. (2018)	USA	Prisoners of both sexes	1399	DSM-3 DSM-4	7.3	yes
Cramer (2016)	Norway	Prisoners of both sexes	857	SCID-II DSM-4	9.4	yes
Czubak and Gawda (2020)	Poland	Male prisoners	314	SCID-II DSM-4	4.80 *	no
Roberts and Coid (2010)	UK	Prisoners of both sexes	496	SCID-II DSM-4	3.19 *	no
Blackburn and Coid (1999)	UK	Violent offenders	164	DSM-3	55	no
van Buitenen et al. (2020)	Netherlands	Mentally ill prisoners	5257	DSM-4/DSM-5	0.9	yes
Brazão et al. (2015)	Portugal	Prisoners of both sexes	294	DSM-4 IPDE	12.2	no
Arroyo and Ortega (2009)	Spain	Prisoners of both sexes	793	ICD-10	2.0	yes
Flórez et al. (2019)	Spain	Prisoners of both sexes	210	ICD-10	21.08	no
Sabareanu and Gonta (2023)	Romania	Maximum-security prison, male prisoners	60	SCID-II DSM-4	35	no
Apostolopoulos et al. (2018)	Greece	Prisoners of both sexes	308	PDQ-4 DSM-4	7.8	no
El-Gilany et al. (2016)	Egypt	Prisoners of both sexes	1350	SCID-II DSM-4	0.1	yes
Kugu et al. (2008)	Turkey	Prisoners/murders	521	SCID-II	4.3	no
Ngunjiri and Waiyaki (2025)	Kenya	Prisoners of both sexes	325	DSM-3-R MCMI-IV	68.8	no

Abbreviations: M—male, F—female, * NPD—narcissistic personality disorder. Assessment of NPD traits, means of NPD traits, not NPD frequency: (a) urban French version, (b) urban Mooré version, (c) rural Mooré version. MCMI-IV (the Millon Clinical Multiaxial Inventory).
